# A Rare Case of Primary Musculoskeletal Hydatid Disease Involving the Deltoid Muscle and Humerus

**DOI:** 10.1590/0037-8682-0279-2025

**Published:** 2025-09-29

**Authors:** M. Alperen Kılıç, Hilal Kırmızıgül, Emre Emekli, Murat Tepe

**Affiliations:** 1 Eskişehir Osmangazi University, Faculty of Medicine, Eskişehir, Turkey.; 2 Eskişehir Osmangazi University, Translational Medicine Application and Research Center, Eskisehir, Turkey.

**FIGURE 2:**
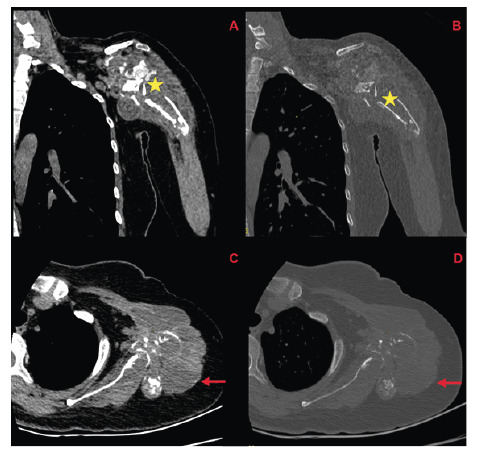
In the patient’s initial shoulder CT scan, bone destruction of the humeral head is observed in both soft tissue and bone windows (**yellow asterisks**). At this level, the bony cortex is not visualized, and the humeral morphology is severely distorted. In the coronal and axial soft tissue windows, soft tissue masses extending within the muscle planes are identified (**red arrows**).

**FIGURE 3:**
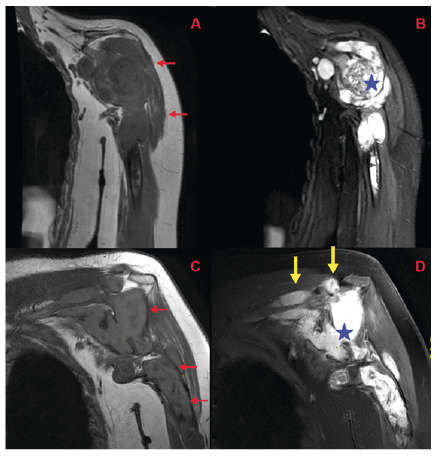
Coronal T1-weighted and PD images at diagnosis **(A and B)** and corresponding coronal oblique T1-weighted and PD images after 10 years of follow-up **(C and D)** demonstrate progression of bone-based lesions despite routine treatment (**red arrows**). Soft tissue involvement also progresses, affecting the joint at the humeral head (blue asterisks). In the current imaging, lesions are more clearly identifiable within the supraspinatus and deltoid muscle planes (**yellow arrows**).

A 38-year-old woman presented with pain in her left shoulder upon palpation and during movement. Laboratory tests showed normal C-reactive protein (CRP) levels and sedimentation rates; however, ELISA results were suspiciously positive for hydatid cysts. Ultrasonography revealed a thick-walled, septate, anechoic cystic lesion. Contrast-enhanced shoulder magnetic resonance imaging revealed lesions extending from the humeral head to the elbow joint, causing proximal bone destruction and distal multilocular cystic lesions with internal septation within the bone marrow **(** Figures 1 and 2 **)**. Similar cystic lesions were found in the deltoid and supraspinatus muscles as well as in other muscle layers and subcutaneous tissue around the shoulder. Aspiration biopsy revealed acellular germinative membrane-like structures consistent with hydatid cysts. The patient was managed with aspiration and followed-up for 10 years, during which the lesion showed minimal progression but remained stable **(** Figure 3 **)**. At the most recent visit, the patient experienced recurring shoulder pain and movement restriction; the CRP level was elevated to 241 mg/L, while other laboratory parameters remained normal. Hydatid cysts primarily cycle between dogs and can infect humans through exposure to contaminated food and water^1^. They are found worldwide, particularly in the Mediterranean region. Musculoskeletal involvement is rare, accounting for 0.5-5% of cases and is almost always secondary to liver or lung infection^2^. Primary muscular hydatid cysts are uncommon and typically appear in peripheral muscles, such as the supraspinatus, biceps brachii, pectoralis major, gracilis, psoas, sartorius, and quadriceps. Few cases of hydatid cysts in the deltoid muscle have been reported in the literature^3^.

**FİGURE 1:**
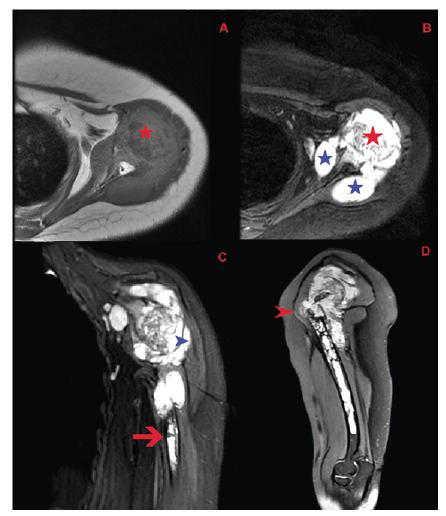
In the patient’s initial shoulder MRI , axial images show an intramedullary lesion within the humeral head causing bone destruction (red asterisks). **A.** On T1-weighted images, the lesion demonstrates intermediate signal intensity similar to muscle. **B.** On PD images, it appears hyperintense. An increased amount of reactive fluid is observed in the glenohumeral joint space (blue asterisks). **C.** On coronal PD images, the lesion extends distally along the humeral diaphysis (red arrow). In addition, lesions with a multicystic nature and internal septations are observed within adjacent muscle compartments, suggesting muscular involvement (blue arrowhead). **D.** On sagittal PD images, a pathological fracture is seen at the humeral head with disruption of the axis at this level (red arrowhead).
